# LRP5, Bone Mass Polymorphisms and Skeletal Disorders

**DOI:** 10.3390/genes14101846

**Published:** 2023-09-23

**Authors:** Jake Littman, Wentian Yang, Jon Olansen, Chanika Phornphutkul, Roy K. Aaron

**Affiliations:** 1 Department of Orthopedic Surgery, Warren Alpert Medical School of Brown University, Providence, RI 02903, USA; 2 School of Medicine, University of Pittsburgh, Pittsburgh, PA 15261, USA; 3 Division of Human Genetics, Department of Pediatrics, Hasbro Children’s Hospital, Warren Alpert Medical School of Brown University, Providence, RI 02903, USA

**Keywords:** LRP5, low-density lipoprotein receptor-related protein 5, skeletal dysmorphogenesis, low bone mass, high bone mass, OPPG, osteoporosis-pseudoglioma syndrome, skeletal phenotype

## Abstract

The formation and maintenance of the gross structure and microarchitecture of the human skeleton require the concerted functioning of a plethora of morphogenic signaling processes. Through recent discoveries in the field of genetics, numerous genotypic variants have been implicated in pathologic skeletal phenotypes and disorders arising from the disturbance of one or more of these processes. For example, total loss-of-function variants of *LRP5* were found to be the cause of osteoporosis-pseudoglioma syndrome (OPPG). *LRP5* encodes for the low-density lipoprotein receptor-related protein 5, a co-receptor in the canonical WNT–β-catenin signaling pathway and a crucial protein involved in the formation and maintenance of homeostasis of the human skeleton. Beyond OPPG, other partial loss-of-function variants of *LRP5* have been found to be associated with other low bone mass phenotypes and disorders, while *LRP5* gain-of-function variants have been implicated in high bone mass phenotypes. This review introduces the roles that *LRP5* plays in skeletal morphogenesis and discusses some of the structural consequences that result from abnormalities in *LRP5*. A greater understanding of how the LRP5 receptor functions in bone and other body tissues could provide insights into a variety of pathologies and their potential treatments, from osteoporosis and a variety of skeletal abnormalities to congenital disorders that can lead to lifelong disabilities.

## 1. Introduction

Numerous processes take place within the skeletal system to maintain homeostasis and conserve its role as a protective, supportive, and structural scaffold of the human body. Integral to this goal is the maintenance of adequate bone mineral density (BMD), which is mostly dependent on a finely tuned balance between bone formation by osteoblasts and bone degradation by osteoclasts. Also of utmost importance is the establishment and maintenance of the shape and structure of bone which relies on a myriad of physical and chemical stimuli promoting pattern formation in development, the growth of the juvenile skeleton, and remodeling in response to stress in the adult skeleton. Given the complexity and sheer number of factors that affect skeletal growth, formation, and maintenance, it is not surprising that there is a myriad of pathologies in which aberrations in one or a combination of these processes result in malformations and dysplasias.

The low-density lipoprotein receptor-related protein 5 (LRP5; OMIM 603506 [1]) was first implicated in skeletal pathology in 2001 when it was determined that autosomal recessive loss-of-function pathogenic variants in *LRP5*, the gene encoding for the LRP5 receptor, leads to osteoporosis-pseudoglioma syndrome (OPPG; OMIM 259770 [2]), a disorder characterized by congenital or infancy-onset vision loss and severe osteoporosis [3]. One year later in 2002, it was determined that an autosomal dominant gain-of-function point variant in *LRP5* was the cause of one family’s abnormally high bone mass phenotype without other abnormalities, such as dysmorphogenesis or an increased incidence of fracture [4]. From these studies, and from the growing body of work examining patients with a range of *LRP5* variants and bone mass polymorphisms that has emerged since, a seemingly simple conclusion can be drawn. Gain-of-function and other variants that lead to the increased functional capability of the LRP5 receptor are associated with increased bone mass, and loss-of-function and other variants (including nonsense variants [5,6]) that lead to the decreased functional capability of the LRP5 receptor are associated with decreased bone mass. However, this direct correlation is only part of the clinical picture. Variants in *LRP5* often lead to phenotypic variability aside from changes in bone mineral density, ranging from grossly observed morphogenetic alterations in the axial, appendicular, and craniomaxillofacial skeleton to cellular-level disturbances in the function of osteoblasts, osteocytes, and osteoclasts, which are both discussed in further detail within this report.

In the less than 25 years since loss-of-function *LRP5* variants were first determined to be the causative mechanism of OPPG, many reports have been published identifying *LRP5* variants as the cause of a variety of phenotypic abnormalities and pathologies. This review provides an introduction to the important roles that *LRP5* plays in skeletal morphogenesis and the structural consequences that result from abnormalities in *LRP5*. A greater understanding of how the LRP5 receptor functions in bone and other body tissues could provide insights into a wide variety of pathologies and their potential treatments, from osteoporosis and a variety of skeletal abnormalities to congenital disorders that can lead to vision loss and lifelong disabilities.

## 2. The Structure and Function of LRP5

The *LRP5* gene contains 23 exons, encodes 1615 amino acids, and is located on chromosome 11q13 [7]. The LRP5 protein is largely extracellular, containing a single transmembrane domain and four extracellular β-propeller motifs [8]. There is some evidence that variants in the first propeller are primarily associated with high bone mass phenotypes, while variants in the second and third propellers are mainly associated with low bone mass phenotypes [9]. However, these patterns are being continuously challenged by the discovery of more variants that do not follow these conventions [10,11,12]. The LRP5 protein plays a significant role in the highly conserved canonical WNT signaling pathway, also known as the WNT–β-catenin pathway, which is involved in multiple processes, including cell fate determination, organogenesis, limb pattern formation, injury repair, and the pathogenesis of a variety of diseases [13,14]. In this pathway, WNT proteins bind to a seven-transmembrane-spanning protein called Frizzled using LRP5 or LRP6 as a co-receptor, leading to a variety of downstream effects that ultimately result in the dissociation of the β-catenin destruction complex and the expression of WNT target genes [14,15]. The structures of LRP5 and LRP6 share over 70% homology, and both are single transmembrane receptors with a large extracellular domain and four tandem β-propeller repeats [16]. There is also considerable crossover in the function of LRP5 and LRP6 [16,17], with some data supporting the notion that certain variants in their associated genes can lead to similar pathophysiological phenotypes [18]. However, there are also distinct differences between the two [14,19], and this review will describe abnormalities arising specifically from *LRP5* variants.

A review of WNT signaling and bone homeostasis published in *Nature Medicine* reported that in every mouse model study examined, increased bone mass was observed as a result of increased pathway activation, and decreased bone mass was observed as a result of increased pathway inhibition [20]. It was also reported in the study that WNT–β-catenin signaling plays essential roles in the synthesis and homeostatic-ratio determination of osteoblasts, osteoclasts, and osteocytes in bone. The study further noted, “WNT signaling represses mesenchymal stem cell (MSC) commitment to the chondrogenic and adipogenic lineages and enhances commitment to, and differentiation along, the osteoblastic lineage. Osteoblast and osteocyte WNT–β-catenin signaling also indirectly represses osteoclast differentiation and bone resorption through the increased secretion of osteoprotegerin” [20]. Furthermore, osteocyte-secreted sclerostin acts as an inhibitor of LRP5 and promotes osteoclast differentiation and resorptive activity [21,22], stimulates the apoptosis of osteoblasts [23], and has been called a “master negative regulator of the canonical WNT signaling in bone tissue” [22]. From this collection of evidence, it becomes clear that alterations in the functionality of the LRP5 receptor and subsequent perturbances in the WNT–β-catenin pathway could feasibly alter the ratio of osteoblasts to osteoclasts and thus influence BMD homeostasis in bone. This hypothesis seems to have been validated in at least one mouse model based on the *LRP5* variants discovered in human patients with altered BMD phenotypes [4,8,24].

In addition to the aforementioned roles of the LRP5 receptor and the WNT–β-catenin pathway in bone, a growing body of research has shown that both play a key role in mechanotransduction [25], elucidating another mechanism by which they might affect the formation and remodeling of the skeleton. In multiple studies, *LRP5* knockout mice consistently showed diminished responsiveness to mechanical stimulation [26,27], while mice with knock-in genes for commonly found *LRP5* variants associated with high bone mass phenotypes showed greater osteogenic response to mechanical stimuli [28]. Recently, further mouse studies have suggested that osteocytes are the principal cell types mediating WNT/LRP5-related bone mass modulations and mechanotransduction [29]. This observation, combined with the assertions that osteocyte density is significantly higher in the craniomaxillofacial skeleton compared to the appendicular skeleton and that skeletal remodeling is more prominent in the facial skeleton than elsewhere [30], provides a plausible mechanism for the emphasis on craniomaxillofacial BMD changes and gross morphogenic alterations observed in patients with *LRP5* variant-related high bone mass phenotypes, as discussed later in this report in the section entitled “High Bone Mass Phenotypes Related to LRP5”.

While there is a large body of data supporting the hypothesis that the LRP5 receptor affects bone formation and homeostasis through the canonical WNT–β-catenin signaling pathway, a discussion on *LRP5* and bone would not be complete without shedding light on other studies that have pointed to an entirely different mechanism of the LRP5 receptor’s effect on bone. Since 2008, a body of evidence has emerged supporting the hypothesis that *LRP5* affects bone mass in a WNT pathway-independent endocrine axis involving duodenum-derived serotonin [31,32,33]. This conclusion has been contested, however [34,35], and it is not yet clear how some of these seemingly incongruous results can be reconciled to create a comprehensive picture of how *LRP5* affects the skeletal system.

## 3. Low Bone Mass Phenotypes Related to LRP5

**Figure 1:**
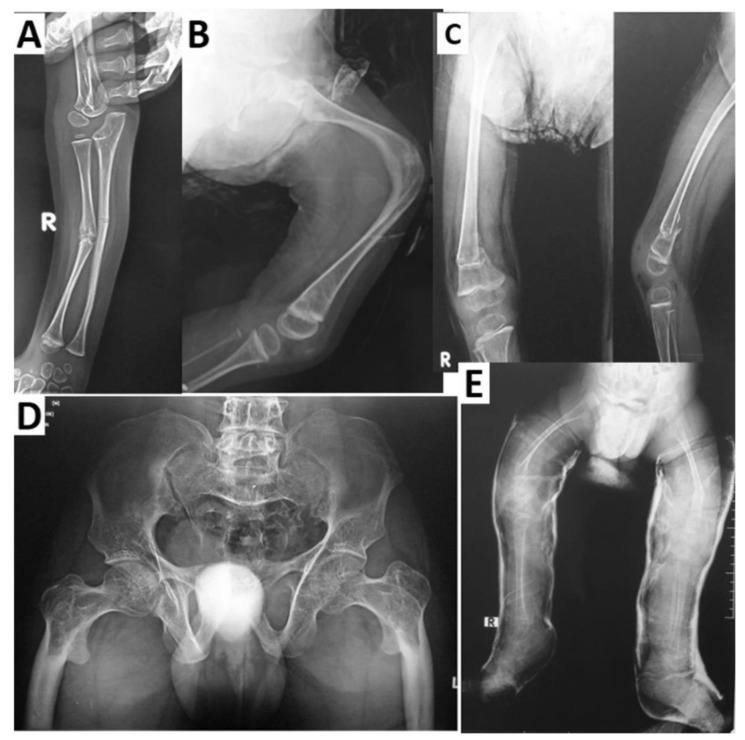
Radiological findings in four patients with OPPG. Multiple fractures and deformities at a young age are typical in OPPG. (**A**) Patient with mid-shaft fractures of the right radius and ulna. (**B**) The same patient displaying an approximately 90-degree angulation deformity in the mid-femoral diaphysis with co-existing deformity of the distal metaphysis. (**C**) Another patient displaying coxa valga and a varus angulated fracture at the distal femoral metaphysis. (**D**) A patient displaying bilateral coxa vara and lytic proximal femora, with disorganized tensile and compressive trabeculae and diaphyseal sclerosis. (**E**) Another patient in bilateral lower extremity casts secondary to bilateral femur fractures exhibiting coxa valga, very thin long bones, and bowing of the femora. Adapted with permission from Ref. [39]. 2022, *Osteoporosis International*.

The first disorder known to be caused by *LRP5* variants was OPPG (OMIM 259770 [2]), which was determined to stem from autosomal recessive loss-of-function pathogenic variants [3]. The skeletal characteristics associated with OPPG include severe osteoporosis, extremity deformities, compressed vertebrae, kyphosis, bowing of the long bones, predisposition to fracture (with many patients suffering multiple fractures from a young age), and thin cortices with enlarged metaphyses (Figure 1) [36,37]. Wormian bone has been observed in at least one case report [38].

Related to OPPG is *LRP5* variant-induced familial exudative vitreoretinopathy (FEVR; OMIM 133780 [40]), a disorder characterized by disorganized retinal blood vessel development leading to incomplete vascularization of the peripheral retina [41,42]. Clinically, FEVR displays genetic heterogeneity, and variants of at least 14 genes have been implicated in its pathogenesis [43]. These include variants in genes related to the WNT signaling pathway, e.g., *LRP5*, *FZD4* (OMIM 604579 [44]), and *CTNNB1* (OMIM 617572 [45]), which have been reported to be responsible for FEVR. Furthermore, while FEVR is usually inherited in an autosomal dominant fashion [46], there is at least one gene, *NDP* (OMIM 300658 [46]), known to cause an X-linked form [47]. Besides regulating retinal vascular development, the genes known to cause FEVR have a diverse and complex relationship with other clinical disorders, for example, Norrie disease, retinopathy of prematurity, and Coats disease [48,49]. Clearly, digenic and multigenic factors are involved in the pathology of FEVR, but decreased bone mass has only been reported thus far in cases related to *LRP5* variants [50]. Based on this, it has been proposed that OPPG and *LRP5* variant-induced FEVR are not actually separate clinical entities and should instead be considered as parts of the same pathophysiologic continuum, with OPPG representing total loss-of-function of the LRP5 protein and *LRP5* variant-induced FEVR representing reduced LRP5 receptor function [42].

**Figure 2:**
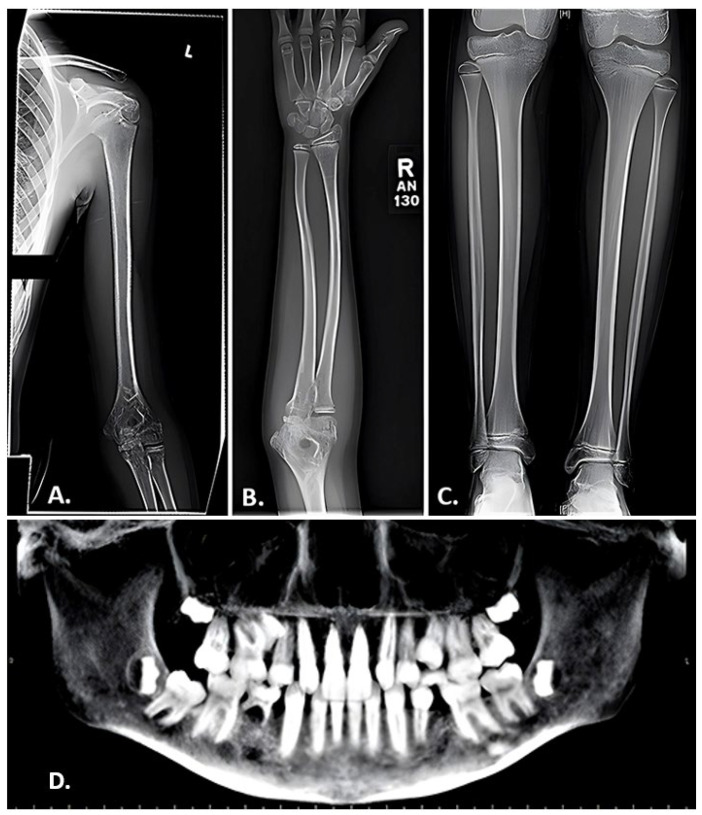
Radiographic findings in a 10-year-old female with FEVR. (**A**,**B**) Anteroposterior view of the left humerus (**A**) and right forearm (**B**) exhibiting gracile bones and thin cortices. (**C**) Anteroposterior view of the tibias revealing endosteal reabsorption with widened medullary canals and thin cortices characteristic of osteoporosis. (**D**) Panorex film displaying impaired dentinogenesis manifested by the absence of the maxillary right and left lateral incisors, maxillary right and left 2nd pre-molars, and right mandibular 1st pre-molar. Adapted with permission from Ref. [51]. 2023. *Orthopedic Research and Reviews* 2023:15 39-45. Originally published by and used with permission from Dove Medical Press Ltd., Macclesfield, England.

**Figure 3:**
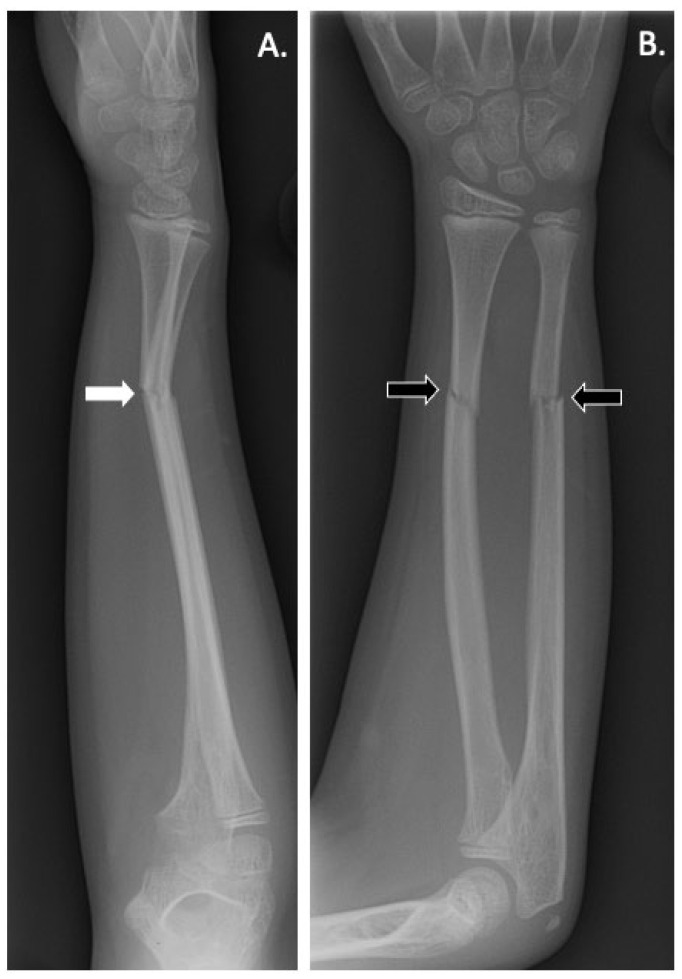
(**A**) Lateral and (**B**) anteroposterior radiographs of the right forearm of a patient with *LRP5* variant-induced familial exudative vitreoretinopathy (FEVR) showing radial and ulnar fractures (black arrows). Angulation is noted on the lateral X-ray (white arrow) suggesting possible instability and loss of physiological position.

**Figure 4:**
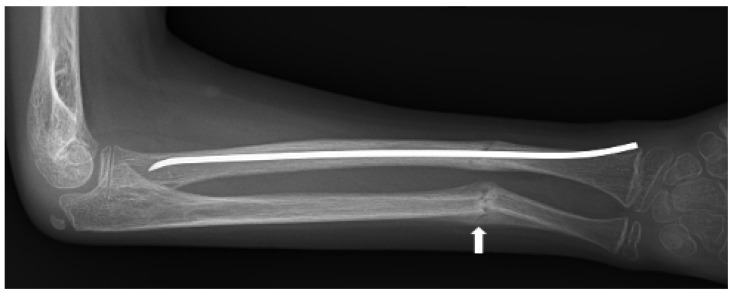
Anteroposterior radiograph of right radial and ulnar fractures of a patient with *LRP5* variant-induced familial exudative vitreoretinopathy (FEVR). The fractures, which were initially undisplaced (Figure 3), became angulated, encroaching upon the interosseous space and potentially limiting supination and pronation. Preservation of rotational motion of the forearm was achieved by intramedullary rodding of the radius, restoring physiological alignment. Fracture callus is seen at the ulnar fracture (white arrow). Due to the young age of the patient, the residual angulation of the ulna underwent remodeling to a physiological position.

The presentation of *LRP5* variant-induced FEVR is extremely variable, with some patients remaining asymptomatic throughout their lifetime and others experiencing multiple fragility fractures from a young age and ocular consequences leading to vision loss or even blindness. Our group previously reported on the skeletal characteristics of two pediatric siblings found to have *LRP5* variant-induced FEVR [51]. Both siblings had significantly reduced bone density, gracile long bones, and widened medullary canals with thin cortices. One sibling was also noted to have coxa valga and the other had impaired dentinogenesis of five adult teeth (Figure 2) [51]. Both also suffered multiple fragility fractures from a young age (Figure 3 and Figure 4). Though our report described aspects of the skeletal phenotype in these patients, there is a need for further research outlining the skeletal phenotypes of other patients with *LRP5* variant-induced FEVR, as much of the current literature focuses on the ocular manifestations of the disorder.

Besides OPPG and FEVR, it has been suggested that loss-of-function *LRP5* variants can lead to other low bone mass disorders, such as juvenile-onset primary osteoporosis [9]. Further work is needed to understand phenotypic variability stemming from different *LRP5* variants. This will hopefully pave the way for targeted therapies for patients affected by these conditions.

## 4. High Bone Mass Phenotypes Related to LRP5

**Figure 5:**
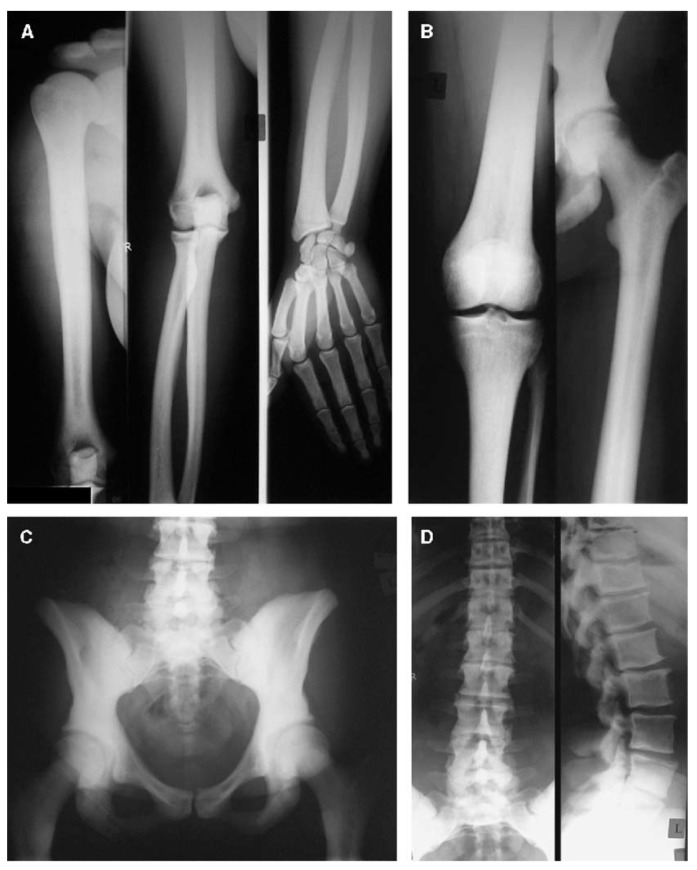
Radiographs of the upper extremity (**A**), lower extremity (**B**), pelvis (**C**), and spine (**D**) in a 31-year-old female with a gain-of-function variant in *LRP5*. Sclerosis can be seen in the vertebrae, the pelvic bones, the sacrum, and the cortices of the long bones, with corresponding narrowing of the medullary canals. Adapted with permission from Ref. [54]. 2005, *Journal of Bone and Mineral Research*.

The first report of a high bone mass phenotype related to *LRP5* was published in 2002, which detailed a family who displayed exceptionally dense bones secondary to a point variant in *LRP5* but were otherwise phenotypically normal [4]. Since then, a body of literature has emerged describing patients with the *LRP5* variant-related high bone mass trait (Figure 5). A 2023 case report and review article by Zhao et al. summarized the clinical characteristics reported for 113 patients with *LRP5*-activating variants (Table 1) [52]. Among the 43 cases they examined for which lumbar spine BMD data were available, 40 patients (93%) had Z-scores greater than 2.5, indicating that they had BMD scores at the 99th percentile compared to age- and sex-matched norms [52]. Furthermore, 51 out of 57 cases (90%) that were assessed for mandible enlargement were found to have this phenotype [52], which is consistent with the previous work conducted by Gregson and Duncan who reviewed the body of literature on patients with *LRP5* variant-related high bone mass phenotypes and also found this to be a prominent characteristic [53].

**Figure 6:**
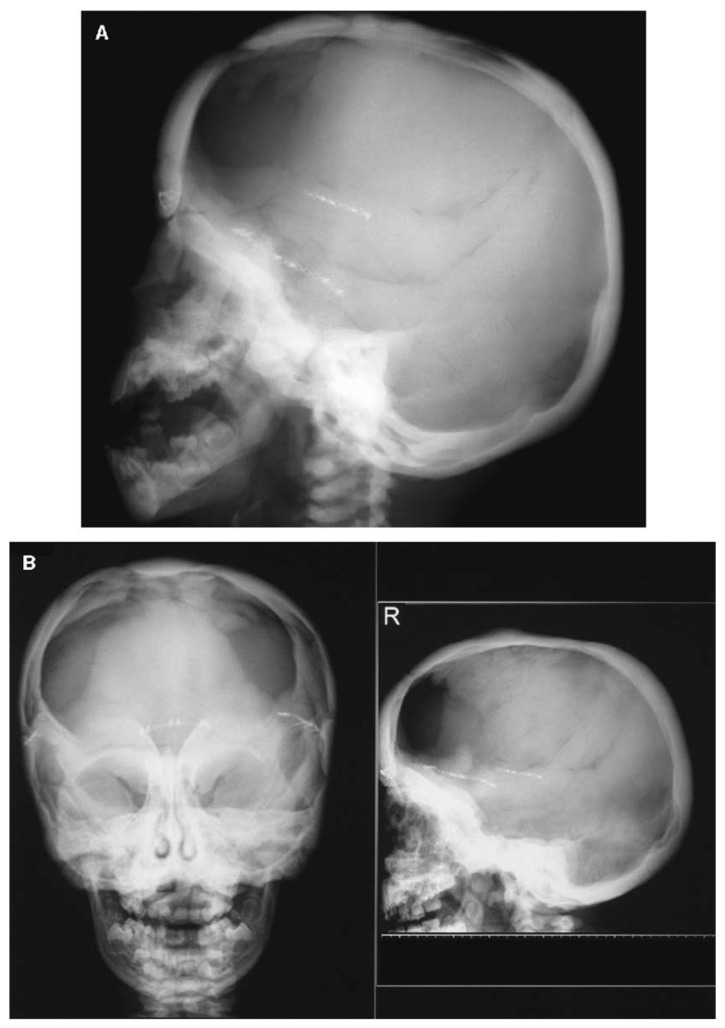
Cranial radiographs of a patient with a gain-of-function missense variant in *LRP5* taken at 1 year and 10 months of age (**A**) and at 3 years and 5 months of age (**B**). Progressive sclerosis of the cranial base and vault can be observed, and a craniotomy was performed at 11 months of age as a treatment for craniosynostosis. Adapted with permission from Ref. [54]. 2005, *Journal of Bone and Mineral Research*.

Diagnoses associated with high bone mass and shown to be related to *LRP5* variants include endosteal hyperostosis, van Buchem disease, and Worth disease [55,56]. Though bearing different names, these disorders are all categorized by high bone mass, reduced fracture risk, and alterations in the first β-propeller domain of the LRP5 receptor, leading to increased activation of canonical WNT signaling [56]. As with *LRP5* variants that lead to low bone mass disorders, the clinical presentation of gain-of-function *LRP5* variants is extremely variable, and patients range from being clinically asymptomatic to having debilitating consequences, such as severe headaches and cranial neuropathies secondary to increased cranial bone mass [18,55,56]. Developmental delay and craniotomy performed as a treatment for craniosynostosis were reported in one family with a gain-of-function missense variant in *LRP5* (Figure 6) [54]. Further work is needed to delineate the diverse genetic variants of *LRP5* and their specific skeletal consequences. This can pave the way for a better understanding of disease processes and potential treatments.

## 5. Altered Bone Mass Due to Genetic Variants in the WNT Signaling Axis Other than LRP5

The WNT signaling pathway is evolutionarily conserved and regulates a wide range of cellular functions by influencing the function of the β-catenin destruction complex. Dysregulation of this signaling complex, through alterations in the LRP5 receptor or a number of other related proteins as discussed below, can be associated with skeletal and extraskeletal manifestations. Examining the changes caused by alterations in related proteins provides greater context for the LRP5 receptor as a key player in the homeostasis of bone.

Axin is one member of the β-catenin destruction complex in which β-catenin can be phosphorylated and targeted for ubiquitin–proteasome degradation. Mammals have two Axin genes, *Axin1* and *Axin2*, whose products are similar in that they are both negative regulators of the WNT–β-catenin signaling pathway [57]. Deletion of *Axin2* in mice has been shown to significantly increase bone mass [58], and variants of the *Axin* genes have been associated with several human malignancies [57], providing evidence that Axin proteins negatively regulate canonical WNT signaling. Indeed, Axin has been shown to have binding sites for proteins involved in WNT signaling, including β-Catenin, GSK-3β, CK1, APC, DVL, and LRP5 [59]. In this protein complex, Axin2/β-catenin signaling eventually targets Bmp2/4, regulating *Osx* expression and controlling the osteogenic differentiation of osteoblast progenitors [58].

Adenomatous polyposis coli (APC) and GSK3 participate in the regulation of β-Catenin turnover. Heterozygous variants and copy number variations of the *APC* gene have been reported to cause increased bone mass in addition to familial adenomatous polyposis [60,61]. Consistent with this finding, GSK3 inhibition with lithium chloride or other compounds has been found to increase bone formation [62]. Similar results were seen in *Lrp5–/–* mice, indicating that LiCl acts downstream of the LRP5 receptor [63]. Disheveled (DVL) is another key component of the WNT signaling pathway. There are three genes that encode for disheveled proteins in this family, *DVL1*, *DVL2*, and *DVL3* [64]. DVL in mice and humans has been proposed to have functional redundancy [64], and therefore mouse models have been used extensively to study its biology. Unlike *Lrp5*−/− mice, *DVL* knockout mice do not display apparent skeletal defects [65]. Instead, overexpression of *DVL1* and *DVL3* has been linked to Hirschsprung’s disease [66].

Sclerostin and Dickkopf-related protein 1 (DKK1), encoded by *SOST* and *DKK1*, respectively, are endogenous WNT signaling antagonists that interact with LRP receptors [67,68]. Variants of *SOST* cause sclerosteosis and van Buchem disease [67,69]. Interestingly, heterozygous carriers of *SOST* variants have increased bone mineral density, suggesting that one affected allele is sufficient to induce a skeletal phenotype and that the effects are dominant [70]. *DKK1* is primarily expressed by osteoblasts and bone marrow mesenchymal stem cells (BMSC) and counteracts the WNT-mediated osteoblastic differentiation of BMSC. *Dkk1*−/− mice die shortly after birth and display developmental head defects and limb dysmorphogenesis [71]. Related to some high bone mass disorders discussed earlier, the SOST protein binds to the first propeller domain of LRP5, and high bone mass variants in *LRP5* have been shown to prevent SOST from binding to LRP5 [72].

## 6. Current and Future Therapeutics Related to LRP5

As WNT-evoked cellular signaling is crucial to bone and other tissue development and homeostasis, it is not surprising that dysregulation of this pathway (by way of either excess promotion or inhibition) due to genetic variants can lead to bone and other organ/tissue pathology. Accordingly, medications that enhance or inhibit cellular signaling along the WNT pathway have therapeutic benefits. Currently, much of the management of *LRP5* variant-induced pathologies is centered around symptomatic treatment and the prevention of further sequelae. In FEVR, for instance, treatment can include preventative measures to decrease the incidence of fragility fractures and laser retinopexy to halt the progression of ocular damage [51]. However, there are some therapeutic agents currently in development stemming from discoveries made through examining the LRP5 receptor and its related pathways. Many of these are aimed at treating osteoporosis by mimicking the increase in canonical WNT signaling seen in patients with gain-of-function *LRP5* variants. The monoclonal antibody romosozumab has been used to prevent vertebral compression fractures in patients with osteoporosis, and functions by blocking the activity of the LRP5 inhibitor, sclerostin, leading to an increase in LRP5-mediated canonical WNT signaling [18,22]. Other strategies currently in development include the creation of chimeric antibodies that can mimic the activity of endogenous WNT ligands and bind to the LRP5 and Frizzled receptors, thus promoting the canonical WNT pathway and increasing bone mass [18].

## 7. Conclusions

The collective body of data on *LRP5* suggests that gain-of-function and other variants that lead to the increased functional capability of the LRP5 receptor are associated with increased bone mass phenotypes and that loss-of-function and other variants that lead to the decreased functional capability of the LRP5 receptor are associated with low bone mass phenotypes. In the complex microenvironments that make up human physiology, direct correlations such as this are invaluable for their potential contributions to a greater understanding of biology and pathophysiology. Though the exact mechanism through which the LRP5 receptor produces its effect on the skeleton is contested, research into its function, including the examination of the canonical WNT pathway, has already brought about beneficial therapeutic tools for patients affected by disorders related to alterations in bone mass, such as osteoporosis. A greater understanding of the LRP5 receptor and related receptors such as LRP6 and Frizzled, as well as their associated roles in modifying WNT/β-catenin signaling and bone homeostasis, could lead to more breakthroughs. More data are needed, however. In vivo and in vitro studies, as well as the collection and reporting of patients affected by conditions in which the function of these proteins is aberrant, all represent further work that could be conducted. These studies have the potential to benefit those affected by the rare genetic diseases caused by *LRP5* variants, as well as many others suffering from more common conditions like osteoporosis.

## Figures and Tables

**Table 1:** Clinical characteristics of patients with *LRP5*-activating variants. Adapted with permission from Ref. [52]. 2023, *Molecular Genetics and Genomics*.

Clinical Characteristic	Percentage of Patients (Proportion) *
Number of cases	113
Mean age at diagnosis (y) (range)	43 (1–83)
Gender	M:F = 54:59
Headache	23.5% (12/51)
Facial nerve palsy	12.5% (7/56)
Hearing loss	16.7% (9/54)
Vision loss	3.8% (2/53)
Bone pain	35.6% (21/59)
Torus palatinus prominence	71.0% (44/62)
Mandible enlargement	89.5% (51/57)
Normal serum ALP levels	88.2% (15/17)
Normal serum P1NP levels	50.0% (3/6)
Normal serum β-CTX levels	66.7% (10/15)
LS BMD Z-score > 2.5SD	93.0% (40/43)
LS BMD Z-score (mean ± SD)	6.2 ± 2.5
TH BMD Z-score > 2.5SD	84.0% (26/31)
TH BMD Z-score (mean ± SD)	5.1 ± 3.1

ALP: alkaline phosphatase; β-CTX: type 1 collagen carboxyl-terminal peptide; P1NP: procollagen type 1 amino-terminal peptide; BMD: bone mineral density; LS: lumbar spine 1–4; TH: total hip; * represents the proportion of patients with the listed clinical feature over total reported cases that measured that feature.

## Data Availability

No new data were created or analyzed in this study. Data sharing is not applicable to this article.
